# Selective Oral Decontamination of the Esophagus to Reduce Microbial Burden in Patients Undergoing Esophagectomy for Esophageal Cancer (SODA)—First Results from a Proof-of-Principle Study

**DOI:** 10.3390/antibiotics14101033

**Published:** 2025-10-15

**Authors:** Johannes Klose, Konrad Lehr, Ulrich Ronellenfitsch, Michelle A. Klose, Daniel Ebert, Artur Rebelo, Alexander Link, Jörg Kleeff

**Affiliations:** 1 Department of Visceral, Vascular and Endocrine Surgery, University Hospital Halle (Saale), Martin-Luther-University Halle-Wittenberg, 06120 Halle (Saale), Germany; ulrich.ronellenfitsch@uk-halle.de (U.R.); joerg.kleeff@uk-halle.de (J.K.); 2 Department of Gastroenterology, Hepatology and Infectious Diseases, Otto-von-Guericke University Hospital, 39120 Magdeburg, Germany; konrad.lehr@med.ovgu.de (K.L.);; 3 Department of Internal Medicine I, University Hospital Halle (Saale), Martin-Luther-University Halle-Wittenberg, 06120 Halle (Saale), Germany; michelle.klose@uk-halle.de; 4 Department of Anesthesiology and Surgical Intensive Care, University Hospital Halle (Saale), Martin-Luther-University Halle-Wittenberg, 06120 Halle (Saale), Germany; daniel.ebert@uk-halle.de; 5 Department of Gastroenterology, Klinikum Bayreuth GmbH, Friedrich-Alexander University Erlangen-Nuernberg (FAU), Medical Campus Upper Franconia, 95445 Bayreuth, Germany

**Keywords:** esophageal cancer, infectious complications, antibiotics, decontamination

## Abstract

**Background/Objectives:** Postoperative pneumonia and other infectious complications after robotic-assisted minimally invasive esophagectomy still contribute to morbidity and mortality. Selective oral decontamination of the esophagus prior to surgery might reduce the rate of infectious complications. However, its impact on the esophageal microbiota is unknown. Therefore, this study aimed to analyze whether selective oral decontamination of the esophagus prior to surgery reduces postoperative pneumonia rates and alters the esophageal microbiome. **Methods:** We conducted a proof-of-principle study including 22 patients who underwent robotic-assisted minimally invasive esophagectomy. Thirteen patients were treated with 50 mg amphotericin B, 8 mg tobramycin, and 10 mg colistin orally 7 days prior to surgery, intraoperatively, and 5 days postoperatively. The remaining nine patients received standard-of-care treatment (no oral decontamination). The esophageal microbiome was assessed using 16S rRNA gene amplicon libraries which were annotated using the Ribosomal Data Project. The incidence of postoperative (at discharge from hospital or 30 days, whichever was later) infectious complications was assessed. **Results:** Selective oral decontamination was associated with reduced overall rates of infectious complications (7.7% vs. 55.5%, *p* = 0.008) and postoperative pneumonia (0% vs. 33.3%, *p* = 0.007). Alterations in the esophageal microbiome depending on selective oral decontamination were detectable. The microbiomes of patients with infectious complications showed higher abundances of *Neisseria* and lower abundances of *Streptococcus* than samples without infectious complications. **Conclusions:** Selective oral decontamination reduced the rate of postoperative complications, postoperative pneumonia in particular, after robot-assisted esophagectomy. Alterations in the microbiome were also evident following decontamination. Further studies with larger sample sizes are necessary to confirm these data.

## 1. Introduction

In the USA, approximately 22,070 new cases of esophageal cancer among men and women are observed per year. Histologically, squamous-cell carcinoma has to be distinguished from adenocarcinoma. Treatment is based on multimodal therapy. Depending on histology and staging, neo-adjuvant chemo- or radio-chemotherapy is applied, followed by surgery [1]. Esophageal resection with intrathoracic anastomosis (the Ivor Lewis procedure) is the surgical treatment of choice. However, although this operation is a minimally invasive procedure which is performed at high-volume centers, it is associated with high rates of postoperative mortality and morbidity [2]. Infectious complications including pneumonia, anastomotic leakage with mediastinitis and pleural empyema, and surgical site infections are the most frequent reasons for prolonged hospitalization and death after surgery [3,4]. Cancer patients with immunosuppression resulting from the underlying disease and from chemo- or radiotherapy are under an increased risk of developing infectious complications. Due to intraoperative single-lung ventilation, development of atelectasis, and compromised postoperative coughing and swallowing, as well as silent aspiration, up to 40% of patients suffer from pneumonia postoperatively and have to be treated with antibiotics or antimycotics [5]. Prolonged durations of intensive care and overall hospital stays mean that this complication is not only a medical problem, but also has an associated socioeconomic impact [6]. Anastomotic leakage occurs in up to 25% of patients after esophagectomy with intrathoracic anastomosis [7,8]. This can cause life-threatening complications, including mediastinitis, pleural empyema, sepsis, and bronchial erosion. Treatments include reoperation, endoluminal vacuum sponge or stent therapy, and resection of the anastomosis with formation of an esophageal stoma. All patients with anastomotic leakage need extensive antibiotic and antimycotic treatment [7,9]. The occurrence of anastomotic leakage prolongs patients’ hospital stays, increases hospital costs, and has a sustained negative impact on patients’ quality of life [10]. Moreover, there is evidence that anastomotic leakage is associated with poorer oncological outcomes [11]. In summary, prevention of infectious complications after esophagectomy is essential for patients’ survival and quality of life.

It is well acknowledged that microbiota from the upper gastrointestinal tract are associated with infectious complications after esophagectomy [12]. Selective decontamination of the digestive tract might alter the esophageal microbiome, resulting in decreased rates of postoperative complications. However, no data from randomized controlled trials are available to support this hypothesis. Therefore, the aim of this study was to show a proof of principle regarding the effects of selective oral decontamination (SOD) of the esophagus on the esophageal microbiome and postoperative infectious complications in adult patients undergoing elective esophagectomy due to esophageal cancer.

## 2. Results

### 2.1. Patient Characteristics

A total of 22 patients were included in the study. Table 1 summarizes the patients’ baseline characteristics. Thirteen patients received selective decontamination of the esophagus prior to surgery. Nine patients were treated with standard of care; these patients served as control. There were no reported side effects of the study medication. The baseline characteristics of age, gender, ASA score, and concomitant diseases were comparable, but the proportion of smokers was higher among patients receiving oral decontamination (Table 1).

Overall rates of infectious complications were 7.7% and 55.5% in patients with and without selective oral decontamination of the esophagus (*p* = 0.008). None of the patients who received selective oral decontamination developed postoperative pneumonia. Anastomotic leakage was observed in one patient (7.7%). In this case, the patient underwent a reoperation and the anastomosis was oversewn.

Three of the patients without selective oral decontamination developed postoperative pneumonia (33.3%). *Airway culture results and representative microbiological analyses were not applicable for all patients.* Two patients suffered from anastomotic leakage (22.2%). In one of these patients the anastomosis was oversewn. The other patient was treated conservatively with an ENDO-sponge and stent placement. There was no mortality in either group.

### 2.2. Microbiome Analysis

**Figure 1:**
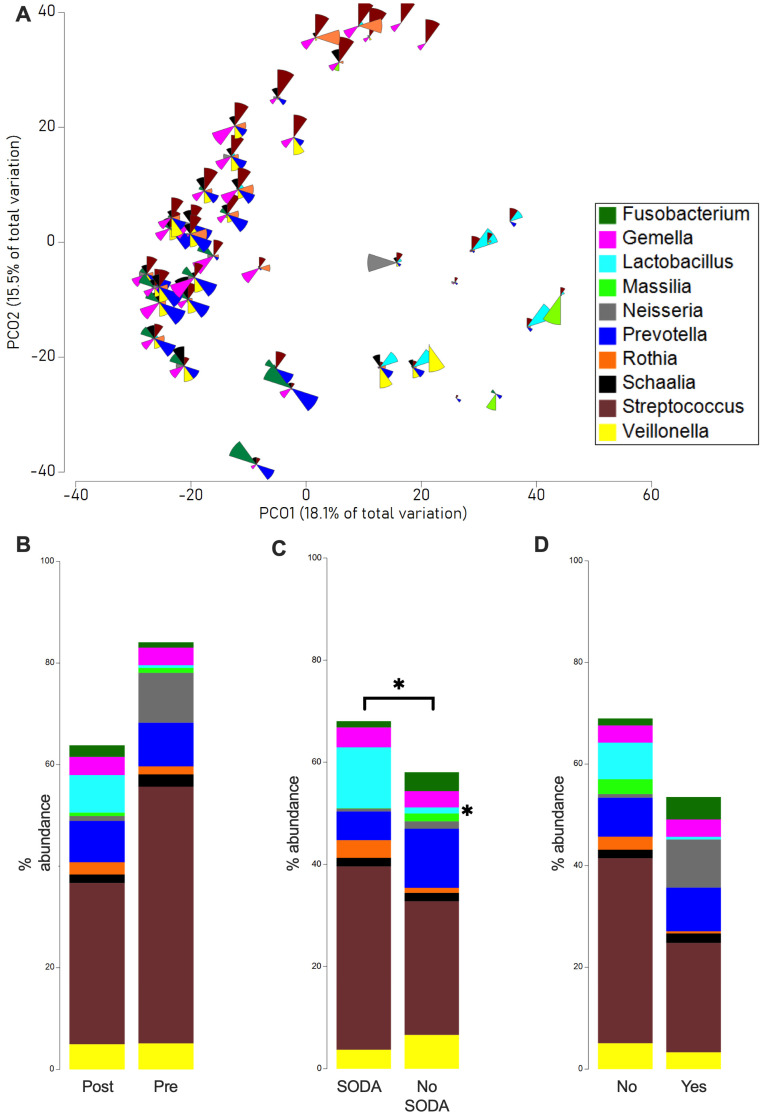
**Microbial changes in SODA-treated samples.** PCO underlying a Bray–Curtis resemblance measurement at genus level, with bubble plot representing the abundance of genera across the sample (**A**). Comparison of the average relative abundance of the 10 most abundant bacteria in sample before and after surgery (**B**), with and without SODA preparation (**C**), and with occurrence and non-occurrence of complications after surgery (**D**). The PERMANOVA test was used for overall microbial community differences and the Mann–Whitney U test for specific taxa abundance differences (* 0.01 < *p* < 0.049).

PCO analysis revealed a high degree of variation in the microbial structure, and also that there was no distinct clustering between the different sample groups (samples taken before or during surgery, with or without selective oral decontamination, with or without occurrence of complications after surgery; possibly due to the limited sample size in this proof-of-principle study. However, after adjusting for smoking, PERMANOVA testing revealed significant differences in overall microbial community structure between patients with or without SODA (*p* = 0.03). A bubble plot revealed that esophageal samples contained typical upper gastrointestinal bacteria (Figure 1A) [13]; the majority had a high abundance of *Streptococcus*, *Prevotella,* or *Gemella*. Other bacteria, such as *Rothia* or *Neisseria*, were present only sporadically, but in high abundance.

Further analysis of bacterial distribution among the groups showed that the samples from before surgery had a higher abundance of *Streptococcus* (pre-surgery: on average 50% from 12% to 88%, post-surgery: on average 31% from 0% to 94%, *p*-value: 0.47) and *Neisseria* (pre-surgery: on average 10% from 0% to 66%, post-surgery: on average 0.9% from 0% to 5%, *p*-value: 0.41, Figure 1B). Samples with and without selective oral decontamination had comparable microbiomes, but samples from patients with selective oral decontamination had a higher abundance of *Lactobacillus* (SODA: on average 11% from 0% to 74%, no SODA: on average 1% from 0% to 16%, *p*-value: 0.02) and a lower abundance of *Prevotella* (SODA: on average 6% from 0% to 23%, no SODA: on average 11% from 0% to 27%, *p*-value: 0.05, Figure 1C). Finally, samples from patients with complications following surgery had a higher abundance of *Neisseria* (complication: on average 9% from 0% to 66%, no Complication: on average 0.8% from 0% to 5%, *p*-value: 0.2) and a lower abundance of *Streptococcus* (complication: on average 21% from 3% to 61%, no Complication: on average 38% from 0% to 94%, *p*-value: 0.1) than samples from patients without infectious complications (Figure 1D). Overall, there were various alterations between the different groups, though most of these did not reach a significant level.

## 3. Discussion

Despite recent advances, esophageal surgery remains associated with high postoperative morbidity rates, mainly due to infectious complications, and, consequently, with impaired long-term survival [14]. In the last decade, minimally invasive esophagectomy has become more and more widespread, and RAMIE has been implemented as a standard surgical technique at many centers [15]. Compared to open or hybrid surgery, RAMIE is associated with fewer postoperative complications, leading to a higher probability of a textbook outcome after esophagectomy [15,16,17]. However, even when RAMIE is applied, infectious complications such as pneumonia and anastomotic leakage still occur in up to 20% of patients [15]. These data indicate that further strategies in addition to optimization of surgical technique are necessary to improve postoperative outcomes in patients with esophageal cancer.

In this pilot study, we observed that selective oral decontamination of the esophagus prior to surgery resulted in a reduction in infectious complications. This may be related to alterations in the esophageal microbiome, although the distinct underlying mechanism cannot be provided in this pilot study. Moreover, the microbiomes of patients suffering from complications after esophagectomy were found to differ from the microbiomes of patients without complications.

Selective oral decontamination of the gastrointestinal tract has previously been reported to reduce the rate of infectious complications after colorectal surgery [18]. Preoperative treatment with oral antibiotics is associated with lower re-intervention rates and improved long-term survival after colorectal resection [19]. It is well acknowledged that microbiota from the upper gastrointestinal tract are associated with infectious complications after esophagectomy [12]. Selective oral decontamination of the esophagus prior to surgery and its impact on patient outcome and occurrence of infectious complications has been reported in several studies [20,21,22]. The selective oral decontamination applied in this study contained three oral, non-absorbable antibiotics and antimycotics, namely, colistin, tobramycin, and amphotericin B. All these drugs have been used for decades, either for selective antimicrobial treatment or in combination for selective decontamination of the digestive tract [23]. Previous reports have described beneficial effects for patients on intensive care units without any increased incidence of adverse effects related to the medication [23,24,25,26]. Additionally, a decreased rate of postoperative complications and anastomotic leakage in gastrointestinal surgery was described in [18]. Two rather historical studies reported reductions in Gram-negative microbiota in the esophagus and in postoperative complications after esophagectomy [22,27]. Although the clinical evidence of reduced postoperative infectious complications after esophagectomy is obvious, the underlying mechanism is unknown and has not been investigated so far. Therefore, this proof-of-principle study aimed to analyze the underlying mechanism of the beneficial effect of selective oral decontamination of the esophagus in reducing postoperative infectious complications. Growing evidence suggests a pivotal role for microbiota in the esophagus as a driver for pneumonia, anastomotic leakage, and other infectious complications [4,28]. The main source for those microbiota is the oropharynx and upper gastrointestinal tract [12]. Recently published data show that Enterococcus and Candida species are predominantly responsible for infectious complications after esophageal resection [29].

We were able to demonstrate that decontamination of the esophagus reduces postoperative infectious complications, a finding in line with previously presented data. Strikingly, we observed that oral anti-infective treatment of the esophagus resulted in alterations in the esophageal microbiome in comparison with non-treated patients. Additionally, infectious complications after esophagectomy were associated with changed abundances of esophageal microbiomes. Similar data were found when the microbiomes of patients with or without anastomotic leakage after colorectal surgery were analyzed [30]. So far, studies on infectious complications after esophagectomy and the esophageal microbiome are missing; however numerous data show that distinct patterns of the esophageal microbiome are associated with the presence of Barrett’s dysplasia and the evolution to esophageal cancer [31,32,33].

The major limitation of this pilot study is the number of patients included in the trial. Therefore, the results have to be interpreted cautiously. The low number of patients might have resulted in a randomly inflated proportion of infectious complications in the group of patients treated with standard of care. Despite this limitation, we showed the protective effects of selective oral decontamination of the esophagus prior to surgery.

## 4. Materials and Methods

### 4.1. Patient Enrolment

For this trial, no formal calculation of sample size was carried out. Instead, convenience sampling of patients who underwent esophagectomy for squamous-cell carcinoma or adenocarcinoma of the esophagus between February 2023 and January 2024 was performed. Trial participation was offered consecutively to all eligible patients during the study period. All patients provided informed consent prior to the initiation of trial-specific procedures.

Neo-adjuvant radiotherapy or chemotherapy was not part of this trial which was administered based on decisions made by multidisciplinary team (MDT) tumor boards.

The ethics committee of the medical faculty of Martin Luther University Halle-Wittenberg approved the present study (#2022-44). The trial was conducted in accordance with the ethical principles of the Declaration of Helsinki and the principles of Good Clinical Practice [34].

### 4.2. Treatment

The trial medication was applied as a mixed solution (Selective Decontamination of the Digestive Tract, SDD), as previously described [20]. The solution contained per 100 g conserved water NRF S.6 (carrier solution) 1.2175 g tobramycin sulfate, 1.18 g colistin sulfate, and 5 g amphotericin B in the form of *Ampho-Moronal suspension*. The trial medication was prepared by the pharmacy of the University Hospital of Halle. The study participants received per dosage 50 mg amphotericin B, 8 mg tobramycin, and 10 mg colistin. The daily oral administration prior to surgery was divided into 7 single doses, each containing 10 mL. Intraoperatively, two further doses of 10 mL were administered via a naso-gastric tube after formation of the gastro-esophageal anastomosis. Administration via the naso-gastric tube was continued for 5 postoperative days and then terminated.

Treatment was started seven days in advance of the operation. All patients underwent upper endoscopy, including biopsy of healthy esophageal mucosa and the tumor region, to assess the naïve esophageal microbiome. The endoscopy was embedded into routine staging prior to surgery, and took place prior to selective decontamination in patients who received the intervention.

Surgery was performed using highly standardized procedures such as robot-assisted minimally invasive esophagectomy (RAMIE) with the *daVinci Xi* system (Intuitive Surgical, Sunnyvale, CA, USA). The guiding surgical concept was two-field lymphadenectomy with stapled intracorporal esophago-gastric anastomosis, as previously described [35]. Repeat microbiome analysis was performed from a biopsy of healthy esophageal mucosa and the tumor region taken from the resection specimen. All operations were performed by two experienced upper-GI surgeons. A chest drainage placement was routinely performed during surgery. After surgery, all patients were transferred to the intensive care unit where rapid extubation was performed and continuous positive airway pressure (CPAP) was applied, four times a day, starting on postoperative day one and continuing until day three.

Patients with an initially uneventful postoperative course were transferred to the intermediate care unit on postoperative day three.

Starting from postoperative day three, in cases of unsuspicious output of less than 300 mL/24 h, the chest drain was removed.

The intraoperatively placed naso-gastric tube was left in place until the fifth postoperative day. Then, in cases where the course was uneventful and infectious parameters were decreasing, oral food uptake was started after removal of the naso-gastric tube. Patients were transferred to a normal ward starting from postoperative day five.

**Figure 2:**
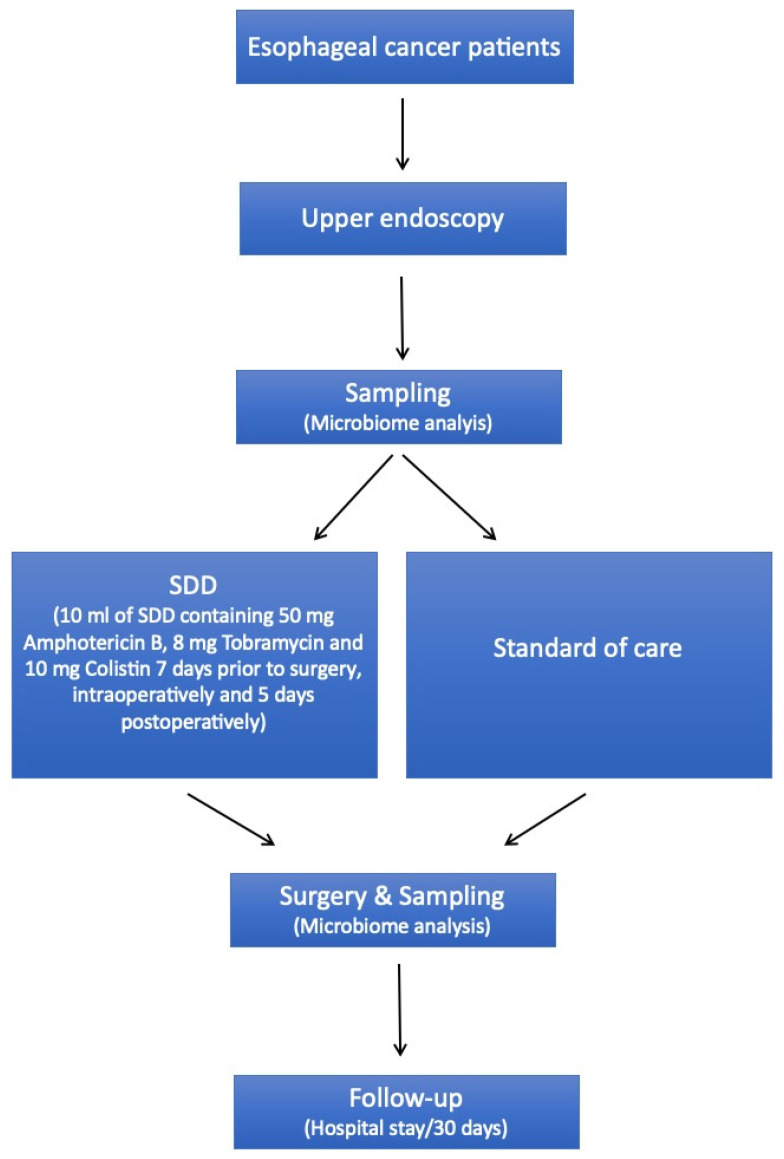
Study protocol.

All patients underwent physiotherapeutic exercises starting from the first postoperative day. After termination of the study treatment, subjects were followed up until the 30th day after surgery. The treatment protocol is summarized in Figure 2.

### 4.3. Microbiome Analysis: DNA Extraction, Sequencing, and Bioinformatics

DNA was extracted from frozen biopsy samples as previously described [30]. In brief, biopsies were lysed in 1 mL of lysis buffer composed of 100 mM Tris-HCl pH 8.0, 100 mM EDTA, 100 mM NaCl, 1% (*w*/*v*) polyvinylpyrrolidone, and 2% (*w*/*v*) sodium dodecyl sulphate in a 2 mL Lysing Matrix E tube (Qbiogene, Alexis Biochemicals, Carlsbad, CA, USA). Additional mechanical lysis was then performed using a FastPrep-24 Instrument (MP Biomedicals, Santa Ana, CA, USA) at a speed of 6.0 m s^−1^ for 40 s. The subsequent DNA extraction was based on phenol/chloroform. The V1–V2 region of the 16S rRNA gene was amplified using the 27F and 338R primers to generate amplicon libraries, employing 40 PCR cycles due to the low bacterial load on the esophageal mucosa. Negative controls were included to monitor for potential artifact introduction. Paired-end sequencing was conducted on a MiSeq with 300 bp (Illumina, San Diego, CA, USA) [36]. After demultiplexing, the fastQ files were analyzed using the dada2 package (version 1.24.0) and R Statistical Software version 4.2.1 (2022; R Foundation for Statistical Computing, Vienna, Austria) [37]. The resulting unique count table was resampled to the size of the smallest library using the Phyloseq package (v1.40.0) [38]. Rarefaction curves were evaluated post-rarefaction to ensure that the data remained representative of the underlying microbial communities (vegan package v2.6-4; https://cran.r-project.org/web/packages/vegan/index.html; accessed date: 21 March 2023). Taxonomic annotation of sequence reads was performed using the Ribosomal Database Project via the rRDP (v1.30.0) and rRDPData (v1.16.0) packages in R [38,39,40]. Microbial communities were analyzed at the taxonomic rank of genus in relative abundances, expressed as percentages. PCO analysis and PERMANOVA testing were performed using the Primer 7 software (PRIMER-E, Auckland, New Zealand) with the PERMANOVA+ add-on package (v7.0), based on a Bray–Curtis resemblance measurement at the genus level. The same software was used to visualize staple plots. The Mann–Whitney U test was used to evaluate statistically significant differences in the distribution of genera across a priori defined groups, utilizing Prism 9.2 software (GraphPad Software, Boston, MA, USA).

### 4.4. Statistical Analysis

Continuous variables were expressed as arithmetic mean and standard deviation (SD) or median with interquartile range (IQR). Student’s two-sided *t*-test was used to compare continuous variables. Categorical data were presented as absolute numbers and relative frequencies, and were compared using the *χ*^2^ test or *Fisher’s exact text, as appropriate*. *p* < 0.05 was considered statistically significant.

### 4.5. Trial Registration

The trial was registered at the German Clinical Trials Register (DRKS)—DRKS00028553 (date of registration: 21 February 2023).

## 5. Conclusions

By demonstrating changes in the microbiomes of patients after decontamination and their impact on the occurrence of infectious complications, we obtained first evidence for the biological effect of oral decontamination. Further studies involving larger numbers of patients, ideally with a randomized controlled design, are necessary to (i) confirm our data and (ii) show the benefits of selective oral decontamination prior to esophageal resection.

## Figures and Tables

**Table 1:** Characteristics of all patients included, classified by preoperative treatment according to the study protocol.

	Patient Characteristics	%		%	*p*-Value
	SODA (n = 13)		No SODA (n = 9)		
**Age (years, mean, IQR)**	62 (54–74)		68 (39–83)		0.135
**Gender**					0.667
Male	12	92.3	8	88.9	
Female	1	7.7	1	11.1	
**ASA Score**					0.942
II	2	15.4	2	22.2	
III	11	84.6	7	77.8	
**BMI (kg/m^2^, mean, IQR)**	25.3 (17.0–36.3)		25.4 (22.1–30.1)		0.93
**Reflux disease**					0.27
Yes	3	23.1	4	44.4	
No	10	76.9	5	55.6	
**Barrett dysplasia**					0.35
Yes	5	38.5	1	11.1	
No	8	61.5	8	88.9	
**Smoking**					**0.032**
Yes	10	76.9	3	33.3	
No	3	23.1	6	66.7	
**Alcohol consumption**					0.497
Yes	6	46.2	2	22.2	
No	7	53.8	7	77.8	
**Histology**					0.398
Squamous-cell carcinoma	5	38.5	2	22.2	
Adenocarcinoma	8	61.5	7	77.8	
**UICC Stage**					0.791
II	3	23.1	2	22.2	
IIIa	3	23.1	4	44.4	
IIIb	7	53.8	3	33.3	
**Tumor location**					0.599
Mid	1	7.7	1	11.1	
Distal	12	92.3	8	88.9	
**Neoadjuvant therapy**					0.87
Yes	10	76.9	1	11.1	
No	3	23.1	8	88.9	
**Duration of surgery in minutes (mean, IQR)**	411 (260–532)		422 (373–480)		0.803
**No. harvested lymph nodes (IQR)**	25 (11–38)		23 (13–28)		0.728
**Pneumonia**					**0.035**
Yes	0	0.0	3	33.3	
No	13	100.0	6	66.7	
**Anastomotic leakage**					0.13
Yes	1	7.7	2	22.2	
No	12	92.3	7	77.8	
**Reoperation**					0.436
Yes	2	15.4	1	11.1	
No	11	84.6	8	88.9	
**Re-intubation**					0.09
Yes	1	7.7	2	22.2	
No	12	92.3	7	77.8	
**Length of hospital stay in days (mean, IQR)**	45 (9–92)		58 (15–85)		0.127
**Length of ICU stay in days (mean, IQR)**	6 (1–83)		12 (1–53)		0.119
**ICU readmission**	1	7.7	2	22.2	0.291

ASA—American Society of Anesthesiologists; BMI—body mass index; ICU—intensive care unit; IQR—interquartile range; UICC—Union Internationale Contre le Cancer.

## Data Availability

The data that support the findings of this study are available from the corresponding author upon reasonable request.
